# Shaping the oral microbiota through intimate kissing

**DOI:** 10.1186/2049-2618-2-41

**Published:** 2014-11-17

**Authors:** Remco Kort, Martien Caspers, Astrid van de Graaf, Wim van Egmond, Bart Keijser, Guus Roeselers

**Affiliations:** 1 TNO Microbiology and Systems Biology, Utrechtseweg 48, 3704 HE Zeist, The Netherlands; 2 Micropia, Natura Artis Magistra, Plantage Kerklaan 38-40, 1018 CZ Amsterdam, The Netherlands; 3 VU University Amsterdam, Molecular Cell Physiology, De Boelelaan 1085, 1081 HV Amsterdam, The Netherlands

**Keywords:** Intimate kiss, Oral microbiota, Tongue, Saliva, Next generation sequencing, Streptococcus, *Lactobacillus*

## Abstract

**Background:**

The variation of microbial communities associated with the human body can be the cause of many factors, including the human genetic makeup, diet, age, surroundings, and sexual behavior. In this study, we investigated the effects of intimate kissing on the oral microbiota of 21 couples by self-administered questionnaires about their past kissing behavior and by the evaluation of tongue and salivary microbiota samples in a controlled kissing experiment. In addition, we quantified the number of bacteria exchanged during intimate kissing by the use of marker bacteria introduced through the intake of a probiotic yoghurt drink by one of the partners prior to a second intimate kiss.

**Results:**

Similarity indices of microbial communities show that average partners have a more similar oral microbiota composition compared to unrelated individuals, with by far most pronounced similarity for communities associated with the tongue surface. An intimate kiss did not lead to a significant *additional* increase of the average similarity of the oral microbiota between partners. However, clear correlations were observed between the similarity indices of the salivary microbiota of couples and self-reported kiss frequencies, and the reported time passed after the latest kiss. In control experiments for bacterial transfer, we identified the probiotic *Lactobacillus* and *Bifidobacterium* marker bacteria in most kiss receivers, corresponding to an average total bacterial transfer of 80 million bacteria per intimate kiss of 10 s.

**Conclusions:**

This study indicates that a shared salivary microbiota requires a frequent and recent bacterial exchange and is therefore most pronounced in couples with relatively high intimate kiss frequencies. The microbiota on the dorsal surface of the tongue is more similar among partners than unrelated individuals, but its similarity does not clearly correlate to kissing behavior, suggesting an important role for specific selection mechanisms resulting from a shared lifestyle, environment, or genetic factors from the host. Furthermore, our findings imply that some of the collective bacteria among partners are only transiently present, while others have found a true niche on the tongue’s surface allowing long-term colonization.

## Background

Mouth-to-mouth contact has been observed in a wide variety of animals, including fish, birds, and primates and serves a range of functions, including the assessment of physical abilities and the acquirement of food. However, intimate kissing involving full tongue contact and saliva exchange appears to be an adaptive courtship behavior unique to humankind and is common in over 90% of known cultures, as reported in [1] and references herein. Interestingly, the current explanations for the function of intimate kissing in humans include an important role for the microbiota and viruses present in the oral cavity, although to our knowledge, the effects of intimate kissing on the oral microbiota have never been studied to date.

A recent study on the importance of kissing in human mating situations proposes that the first kiss serves as a useful mate-assessment function and the following for mediation of feelings of attachment in long term relationships, rather than the facilitation of sexual arousal [1]. Kissing may contribute in mate assessment and bonding via sampling of chemical taste cues in the saliva [2], including those resulting from the metabolic activity of the bacterial community on the surface of the tongue.

Hendrie and Brewer hypothesized another advantage for intimate kissing [3]. They argued that information on the quality of a partner can also be obtained from close physical proximity, and that saliva exchange could involve a risk resulting from the exposure to pathogenic microorganisms, leaving mate assessment an unlikely pressure for its development. They postulated that intimate kissing evolved to protect pregnant women against *in utero* teratogenesis by human cytomegalovirus, which is readily transmitted through saliva, urine and semen, and would cause less severe symptoms when acquired prior to pregnancy [3]. However, both functions for intimate kissing, mate assessment or some form of immunization, involve an important role for the viruses and microorganisms that reside in our mouth.

The oral cavity has two main types of surfaces for microbial colonization: non-shedding surfaces (teeth) and shedding surfaces (mucosa), including gingival crevices, tongue, hard palate, soft palate, cheeks, and lips. A number of studies have shown that each of these type of surfaces provide a range of habitats with a characteristic microbiota [4,5]. It has been estimated that the oral cavity harbors approximately a total of 700 different, mostly anaerobic species [4]. Saliva also contains a large number of bacteria, but the existence of a true indigenous salivary microbiota is a matter of debate, as the high flow rate of saliva and low nutrient content would not easily allow bacterial proliferation. To a large extent, the organisms found in the saliva are those shed by or dislodged from other oral surfaces, in particular the dorsal surface of the tongue [5].

In this study, we investigated the effect of intimate kissing on the oral microbiota. A number of factors are important for shaping our microbiota, including genetic relatedness, diet, and age, but also our surroundings, including the individuals with whom we interact. A recent study indicated that household members, particularly couples, shared more of their microbiota than individuals from different households, with stronger effects of a shared household on skin than oral or fecal microbiota [6].

We investigated (i) if kissing partners share a more similar oral microbiota (tongue and saliva) than people with no intimate relationship, (ii) if self-reported kiss frequencies over the last year, the time passed after the latest kiss and the actual act of kissing influences the composition of oral microbiota, and (iii) the number of bacteria transferred by the use of marker bacteria.

We present evidence that partners share part of their microbiota on the surface of their tongues, and for at least hours in their saliva after kissing, suggesting that collective bacteria found a niche for colonization in the oral cavity, some transiently, others permanently.

## Results and discussion

### Composition of the oral microbiota

**Figure 1:**
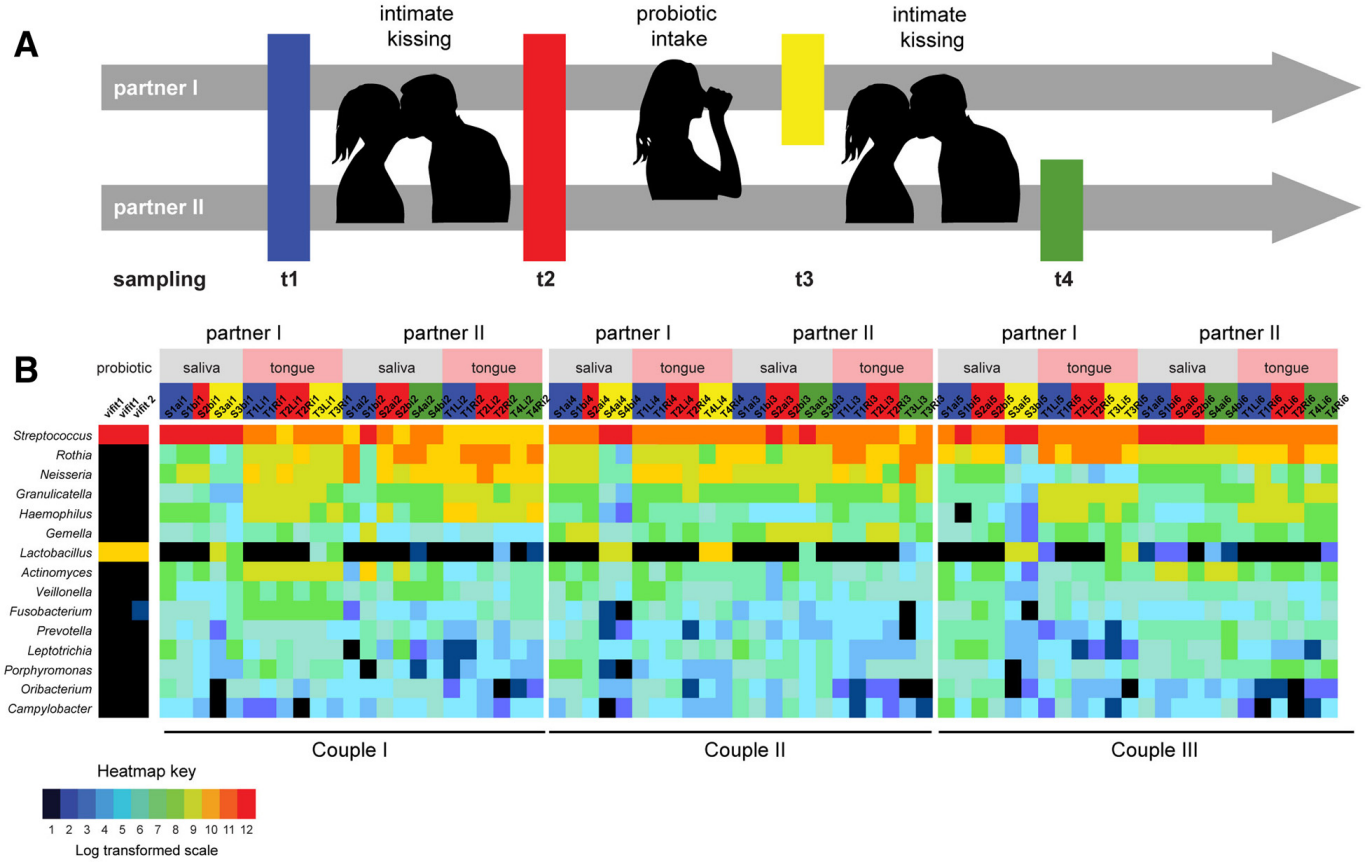
**Study design and top 15 abundant genera of the oral microbiota and probiotic yoghurt. (A)** Samples of both members of recruited couples were collected of the anterior dorsal tongue surface and saliva prior to (blue) and after an intimate kiss of 10 s (red). One of the partners was asked to consume 50 ml of a probiotic yoghurt drink, and again tongue and saliva were collected of the donator prior to (yellow) and the receiver after a second intimate kiss (green). **(B)** Relative abundances of the top 15 most dominant genera of the oral microbiota and probiotic yoghurt plotted on a log transformed color-coded rainbow scale from 0 to 12 from black, blue, green, yellow, orange to red. Headers include partner, probiotic yoghurt drink, saliva, tongue, sample IDs, couples, and sample type, as indicated by the same color-coding in the study design.

In this study, we sampled 21 couples, including one female and one male homosexual couple, according to the scheme depicted in Figure 1A, resulting in 84 tongue and 84 salivary microbiota samples. Three couples were sampled in duplicate, and three probiotic yoghurt drink samples were included, which were subjected to bacterial composition analysis by a single bar-coded amplicon sequencing run. The total amount of reads from the run was 432,089, and the total number of bases was 134,916,296 bp, corresponding to an average read length of 312 bp after quality processing. A total number of 3,000 OTUs, based on 97% sequence percent similarity, were identified in the tongue and saliva samples (Additional file 1). Only 25 of these OTUs were observed in more than 50% of all 284 oral microbiota samples. On average, 96 ± 28 OTUs were observed per oral sample. After taxonomic classification, we depicted the 15 most abundant genera present in the oral microbiota and the yoghurt drink in Figure 1B. The first three columns indicate the genera identified in the probiotic drink, including *Streptococcus* as most abundant, and *Lactobacillus* as less abundant genus. In addition, we identified the genus *Bifidobacterium* (Additional file 1). This is in agreement with the expected presence of yoghurt-producing species *Streptococcus thermophilus* and *Lactobacillus delbrueckii* subsp. *bulgaricus* and the probiotic additives *Lactobacillus rhamnosus* GG, *Lactobobacillus acidophilus* LA5, and *Bifidobacterium lactis* BB12. The top 10 genera of the oral microbiota in our study include *Streptococcus*, *Rothia*, *Neisseria*, *Gemella*, *Granulicatella*, *Haemophilus*, *Actinomyces*, *Veillonella*, *Porphyromonas*, and *Fusobacterium*, all known to be among the predominant genera of the normal microbiota in the oral cavity [5].

### Partners share part of their tongue microbiota

**Figure 2:**
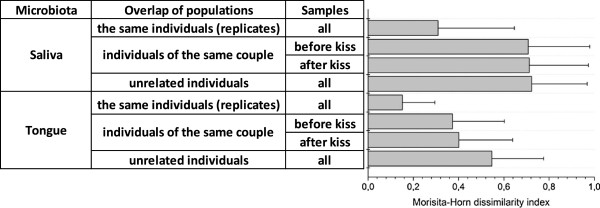
**Average Morisita-Horn dissimilarity indices among samples of the same individuals, individuals within a couple, and unrelated individuals.** Similarity of oral microbiota is indicated by the average Morita Horn’s indices for saliva and tongue of samples from (i) replicates of the same individuals, (ii) the two individuals within a couple before kissing, (iii) the two different individuals within a couple after kissing, and (iv) the different, unrelated individuals.

We investigated the average level of similarity of the tongue and salivary microbiota between multiple samples of a single individual, between couple members, and among the unrelated individuals. Therefore, we calculated similarity indices before and after the kiss of all 21 couples and analyzed their average values (Figure 2). We used the Morisita-Horn (MH index), a commonly applied dissimilarity measure for pairwise comparisons of microbiota within certain groups of populations (see e.g., [7]). The index is expressed in a scale from zero (completely similar) to one (completely dissimilar). Based on the MH indices, the highest degrees of pairwise similarity are observed for the surface of the tongue for replicate samples of the same individuals, showing a value of 0.15. This is more similar than the average index of 0.31 for replicate samples of the salivary microbiota. This is in agreement with the highly dynamic nature of salivary microbiota in the oral cavity *versus* the relatively stable surface-associated microbial community of the tongue’s surface. Apparently, the overall composition of the salivary microbiota changes rapidly over time in a single individual, as replicate samples were collected within a time window of 1 min.

A comparison of microbiota between couple members and unrelated individuals shows that the tongue microbiota is much more similar for couple members, an average MH index value of 0.37 versus 0.55 for unrelated individuals (*p* value of the Wilcoxon rank-sum test =1.4 × 10^−7^), while this does not apply to the salivary microbiota, where we calculated MH index values of 0.71 versus 0.72 for unrelated individuals (*p* value >0.1; the difference is not significant). Comparable results were obtained when other indices for similarities in community structure were used, including the Bray-Curtis index, the Yue and Clayton theta, Species Profile, and Spearman similarity coefficients (Additional file 2).

The shared microbiota on the tongue could be more evident as a long-term effect of couples living together, effectuated through sharing dietary and personal care habits. This finding is in agreement with a recent study, showing that household members, in particular couples, shared more of their microbiota than individuals from different households [6]. In addition, we investigated the effect of intimate kissing on the microbiota similarity. On average, the salivary and tongue microbiota did not change in members of the same couple after an intimate kiss, as the *p* values of the Wilcoxon rank-sum test for these hypotheses were 0.45 and 0.30 for salivary and tongue microbiota, respectively, both considered not significant (*p* values >0.1).

### Similarity of the oral microbiota correlates to self-reported kiss history

We included the self-reported history of intimate kissing behavior in our study and examined correlations between this behavior and all the similarity indices of the oral microbiota. We asked all 21 couples to report their last year’s average intimate kiss frequency and the period of time passed after their latest intimate kiss. We calculated all the average kiss frequencies and average periods past after the latest kiss. Strikingly, 74% of the men reported higher intimate kiss frequencies than the women of the same couple, resulting in a male average of 10 and a female average of five intimate kisses per day (Additional file 3). This probably results from male over reporting, as previously noted in an analysis of self-reports on sexual behavior, including number of partners and frequency of intercourse, in particular among unmarried couples [8]. One report of an average of 50 intimate kisses per day over the last year (Additional file 3) was according to the opinion of the authors unrealistically high, not in agreement with the reported time to latest kiss of 18 h and showed a large discrepancy with the self-reported kiss frequency of his partner of eight intimate kisses per day. Therefore, we excluded the kiss frequency of this couple from the correlation analysis with the kiss frequencies and MH indices in this study.

**Figure 3:**
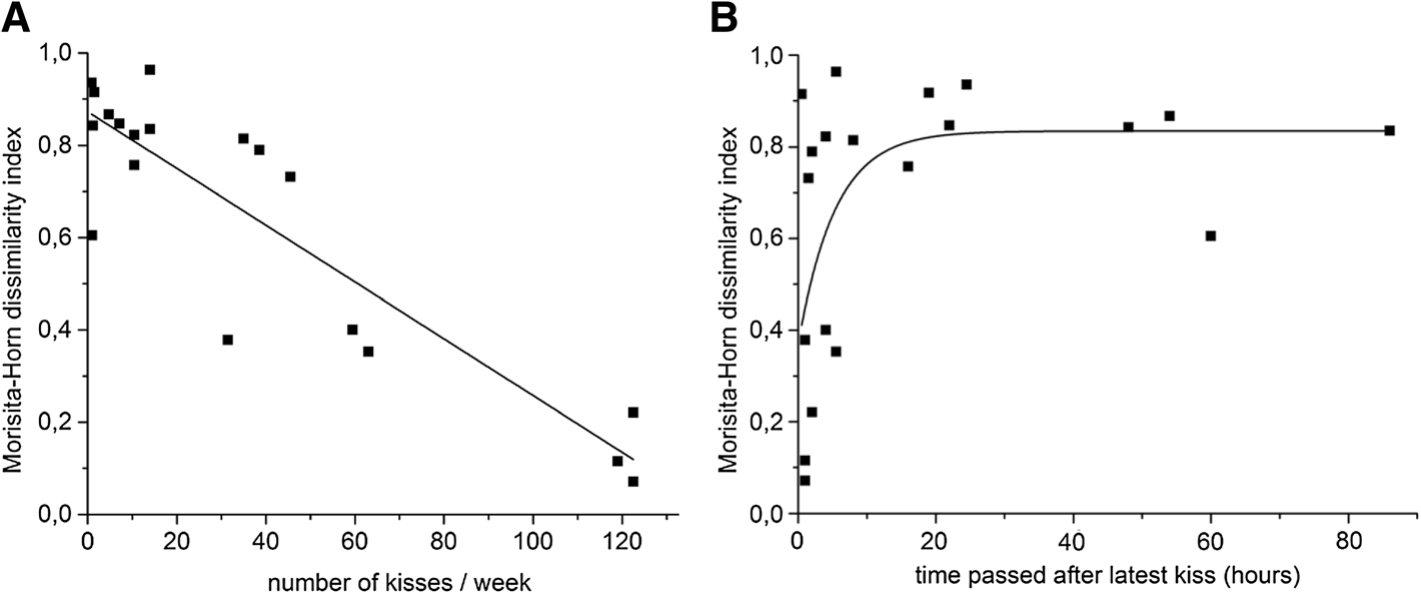
**Correlations between microbiota similarity and intimate kiss history.** Correlation between the Morisita-Horn dissimilarity index of the salivary microbiota from couple members and **(A)** self-reported kiss frequencies or **(B)** self-reported time after the latest kiss.

The dissimilarity indices were plotted as a function of the average self-reported kiss frequencies by males and females of the same couple (Figure 3A). We fitted the data with a linear regression model (R-squared =0.82). The data clearly shows that the salivary microbiota becomes more similar when couples intimately kiss at relatively high frequencies. The linear fit of the data indicates that frequencies of at least 9 kisses per day are required in order to obtain an MH index <0.5. The tongue microbiota does not show a significant correlation with intimate kissing frequency (Additional file 3), in agreement with a transient salivary microbiota and a more permanent tongue microbiota. After a single intimate kiss, we did not observe a significant effect on the similarity of the salivary and the tongue microbiota. This is confirmed in plots of similarity indices of all couples (Additional file 3); we only observed a more similar salivary microbiota in a limited number of couples. In agreement with the data that indicate that a relatively large number of kisses is required to substantially equilibrate the salivary microbiota (at least nine per day), the effect of a single intimate kiss is limited.

We investigated the effect of the time passed after the latest kiss on the similarity index (Figure 3B). Data were fit with an exponential rise function (R-squared =0.24) and indicated that MH index >0.5, if a sample is taken longer than 1 h and 45 min after the latest kiss. Although the coefficient of determination is rather low, the data are consistent with a model that holds that nine intimate kisses per day, and a period of time of less than 1 h and 45 min is required to maintain a substantially equilibrated salivary microbiota (MH index >0.5). There was no correlation between the time passed after the latest kiss and the tongue microbiota and no clear additional effect on this correlation after a single kiss (Additional file 3).

It should be noted that constitutive microbial colonization of saliva is still a matter of debate, as the high flow rate and relatively low content of nutrients do not easily allow bacterial proliferation. The bacteria in the saliva may be mostly shed by or dislodged from the oral surfaces, particularly from the tongue [5]. On average, the unstimulated flow rate of saliva is 0.3 ml min^−1^, while the stimulated flow rate, which contributes as much as 80% to 90% of the average daily salivary production is at maximum of 7 ml min^−1^[9]. The average saliva volume in the mouth is only 0.74 ml [10]. These numbers indicate that almost constant bacterial exchange is required to maintain a shared salivary microbiota. However, we found in our study that ‘only’ nine kisses per day or a time period of less than 1 h and 45 min after the latest kiss are required for a significantly shared salivary microbiota. In order to interpret our data, we assume that the collective bacteria detected in the saliva after a kiss do attach and *transiently* colonize the oral surfaces, in particular the tongue, of the kissing partner. This phenomenon has also been described in a number of studies with probiotic lactobacilli, which reported transient colonization of the surface of the tongue up to a period of 2 weeks after oral intake (see e.g., [11] and reference herein). Possibly, an adapted group of the shared bacterial species colonizes the tongue more permanently, explaining the similarity in the tongue surface microbiota among partners found in this study. The effect of multiple kisses appears more obvious on the variable and more distinct salivary microbiota than the more constant and more similar tongue microbiota. According to this notion, both transient and permanent colonizers play an essential role in shaping the oral microbiota after intimate kissing. As the similarity of tongue microbiota of partners does not depend on the kissing frequency, mouth-to-mouth contact may be essential for the transfer of bacteria, but other covarying factors among partners could contribute to the overall similarity of the oral microbiota, including diet, oral hygiene practices, and dental care. Future studies will aim at the construction of a quantitative model explaining the dynamics of the oral microbiota after kissing and characterization of specific properties of the transient and permanent collective bacteria involved. Visual inspection of the shared microbiota in the saliva and on the tongue (Additional file 4) did not lead to the identification of specific transient and permanent shared genera but indicated that the abundant genera are predominantly shared among partners. The identification of factors that determine the transient or permanent status of bacterial colonizers in the mouth could contribute to the development of novel strategies for the prevention or cure of oral infectious diseases. However, these factors remain to be elucidated and may result from a combination of genetic factors of the host and adaptive mechanisms of the commensal bacteria acquired throughout our lives.

### Amount of bacteria transferred after an intimate kiss

**Figure 4:**
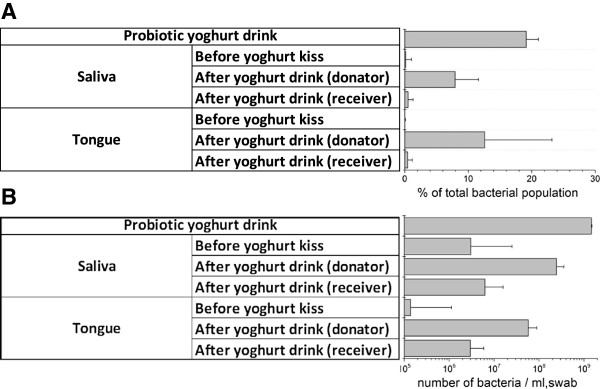
**Estimation of bacterial transfer after an intimate kiss by tracking probiotic marker bacteria.** The OTUs of *Lactobacillus* and *Bifidobacterium* marker bacteria displayed as **(A)** the percentage of the total bacterial population and **(B)** the number of bacteria per ml in the saliva or per swab of the anterior dorsal surface of the tongue.

We evaluated the bacterial transfer by the use of marker bacteria introduced via a probiotic yoghurt drink containing the common yoghurt bacteria *Streptococcus thermophilus* and *Lactobacillus delbrueckii* subsp. *bulgaricus* and the probiotic bacteria *L. rhamnosus* GG, *L. acidophilus* LA5, and *B. lactis* BB12. First, we identified the OTUs in our total data set of 3,000 OTUs, which were linked to the bacteria present in the yoghurt samples. The number of OTUs associated with genera *Streptococcus*, *Lactobacillus*, and *Bifidobacterium* accounted for 99.7% of the bacteria identified in the yoghurt drink samples. As the genus *Streptoccocus* is the most predominant genus in the oral cavity, coinciding with very high backgrounds in the absence of yoghurt (11.1% for saliva and 12.5% for the tongue), we selected the OTUs linked with *Lactobacillus* and *Bifidobacteria*, which constitute 19.1% of the bacteria in yoghurt drink, and on average of 0.15% of the bacteria in the saliva and 0.01% of the bacteria on the tongue. After the yoghurt drink consumption, the levels increased to an average of 7.9% and 12.6% in the donators and after intimate kissing to respectively 0.54% and 0.49% in the receivers (Figure 4A).

The average amount of bacterial DNA was determined for all saliva samples and tongue swabs by 16S rRNA qPCR (Additional file 4) and converted to bacteria per ml or per swab by assuming an average of 2.5 fg DNA per bacterium for all samples [12], as indicated in formula (1) below. This results in averages of 6.4 × 10^9^ bacteria ml^−1^ in the yoghurt drink, 1.8 × 10^9^ bacteria ml^−1^ in the saliva, and 0.8 × 10^9^ bacteria per tongue swab (Figure 4B). Although these values seem relatively high, they are not in disagreement with bacterial concentration reported in previous cultivation-based experiments, which were up to 1.8 10^9^ CFU ml^−1^ for saliva [13] and to be variable on the dorsal tongue surface from 10^7^ to 10^9^ CFU cm^−2^, with the higher densities to the back of the tongue [5]. From the average concentrations and the average marker OTU percentages, we calculated the average transfer by subtracting the values after the kiss from the values before the kiss, assuming that all bacteria transfer with an efficiency equal to that of the marker bacteria (see formula 1 in the ‘Methods’ section). The kiss contact surface in the receiver of the kiss was estimated to cover a total of three tongue swabs and the value for the average saliva volume of 0.74 ml was taken from a previous report [10]. This led to a total average bacterial transfer of 0.8 10^8^ bacteria transferred per intimate kiss of 10 s.

## Conclusions

This study indicates that a shared salivary microbiota requires a frequent and recent bacterial exchange and is most pronounced in couples with relatively high intimate kiss frequencies of at least nine intimate kisses per day or in couples sampled no longer than 1.5 h after the latest kiss. The microbiota on the dorsal surface of the tongue is more similar among partners than unrelated individuals, but its similarity does not clearly correlate to kissing behavior. Our findings suggest that the shared microbiota among partners is able to proliferate in the oral cavity, but the collective bacteria in the saliva are only transiently present and eventually washed out, while those on the tongue’s surface found a true niche, allowing long-term colonization.

## Methods

### Study design and sample collection

In this study, we investigated the effect of intimate kissing on the oral microbiota among human couples visiting the Artis Royal Zoo in Amsterdam on 26 July 2012. A total of 42 individuals (21 couples) between 17 and 45 years old were recruited for this study. After consent for participation in the study, a questionnaire about age, gender, kiss frequency, time passed after the latest kiss, time passed since latest meal, and meal composition was filled out by each individual. As outlined in the experimental set-up in Figure 1A, we sampled the anterior dorsal tongue surface and saliva of both members of each couple before and after an intimate kiss of 10 s. Each individual was asked to donate saliva in a sterile, disposable 15-ml tube. The tongue was swabbed by rotation of a cotton swab over the anterior dorsal tongue surface from left to right. The bacteria on the swab were resuspended in a sterile, disposable 15-ml tube containing 5 ml of sterile physiological salt solution. The saliva and resuspended tongue swabs were frozen instantaneously on dry ice, transported, and stored at −80°C until further processing. Couples were requested to kiss intimately for a period of 10 s, and the saliva and tongue swabs were collected once more. In addition, one of the partners was invited to consume 50 ml of a probiotic yoghurt drink containing *L. rhamnosus* GG, *L. acidophilus* LA5, and *B. lactis* BB12. After 10 s, saliva and tongue swabs were collected from this partner (donator) and after a second intimate kiss of 10 s, saliva and tongue swabs were directly collected from the other partner (receiver). For reproducibility tests, two replicates were collected and analyzed from three different couples.

### Differential interference contrast microscopy

Micrographs of samples of the dorsal surface of the tongue, saliva, and the bacterial content of the probiotic yoghurt drink were obtained by differential interference contrast microscopy (DIC) with a Universal Research Microscope, 63× objective (Zeiss, Oberkochen, Germany) equipped with a DSLR camera (Nikon Inc., Melville, USA). We evaluated oral microbial communities by DIC-microscopy and found a large variety of bacterial morphotypes mostly in aggregates in the salivary and tongue microbiota samples. The micrographs confirm the presence of cocci (*Streptococcus*) and filamentous bacteria (*Actinomyces*) among the predominant members of the oral microbiota. In addition, the DIC micrograph of the yoghurt drink used in this study clearly indicates the presence of cocci (*Streptococcus thermophilus*) and rod-shaped lactobacilli (Additional file 5).

### DNA extraction, amplicon synthesis, and sequencing

The DNA extractions were performed using a phenol bead beating procedure as described previously [14]. Shortly, mechanical disruption of bacterial cells was done by bead beating for 2 minutes in a mini-beadbeater-8 cell disruptor and chromosomal DNA was obtained by binding to and washing from magnetic beads. A quantitative 16S rRNA PCR was performed to determine the amount of bacterial template DNA in the samples as described in [15] and a Bacterial 16S rRNA amplicon library was generated spanning variable regions V5-V7 [16]. The DNA sequences of the amplicon library were determined on a 454 GS-FLX-Titanium Sequencer (Roche, Branford, USA).

### Sequence processing and analysis

The 454 sequence data were demultiplexed and quality filtered as described by Zhao *et al*. [14] using modules implemented in the Mothur software platform [17]. Aligned 16S rRNA gene sequences were clustered into operational taxonomic units (OTUs), defined by 97% identity, using the average linkage clustering method. Analyses of tongue and salivary community similarities (β-diversity) were performed by calculating pairwise distances using the Morisita-Horn dissimilarity index module implemented in Mothur software platform [17]. Taxonomic classifications were performed using the RDP Naïve Bayesian Classifier and the SILVA reference database (release 119).

### Statistical analysis

Probabilities (*p* values) for selected paired differences of data presented in Figure 2 were determined with the non-parametric Wilcoxon rank-sum test and compared to those obtained by the student’s *t* test with one-tailed distribution and two-sample equal variance (Additional file 2). The models used to fit the data in Figure 3A,B included a linear model (*y* = *a* + *bx*) and a non-linear model (*y* = *A*_1_*e*^*x*/*t*1^ + *y*_0_), respectively.

### Quantitative analysis of bacterial transfer

From the average bacterial concentrations (determined by PCR) and the average probiotic marker OTU percentages, we calculated the average bacterial transfer by subtracting the values after the kiss from the values before the kiss, assuming that all bacteria transfer with an efficiency equal to that of the marker bacteria (formula 1).

(1) $$\begin{array}{ll} A & =\;\left[\left(P_{SAK}\right)\left(C_{SAK}K_{C}\right)–\left(P_{SBK}\right)\left(C_{SBK}K_{C}\right)\right]\left[V_{SR}\right]\\ & \;+\left[\left(P_{TAK}\right)\left(C_{TAK}K_{C}\right)–\left(P_{TBK}\right)\left(C_{TBK}K_{C}\right)\right]\left[S_{TR}\right] \end{array}$$

with:

*A* = average amount of bacteria transferred per intimate kiss of 10 s

*C*_SAK_ = average concentration of bacterial DNA in the saliva after kissing in gram ml^−1^

*C*_SBK_ = average concentration of bacterial DNA in the saliva before kissing in gram ml^−1^

*C*_TAK_ = average concentration of bacterial DNA on the tongue after kissing in gram per swab

*C*_TBK_ = average concentration of bacterial DNA on the tongue before kissing in gram per swab

*K*_C_ = average of 0.4 10^12^ bacteria gram^-1^ DNA[12]

*P*_SAK_ = average percentage of marker OTUs in the saliva after kissing

*P*_SBK_ = average percentage of marker OTUs in the saliva before kissing

*P*_TAK_ = average percentage of marker OTUs on the tongue after kissing

*P*_TBK_ = average percentage of marker OTUs on the tongue before kissing

*V*_SR_ = average volume of saliva of the receiver of 0.74 ml [10]

*S*_TR_ = average total kiss contact surface of the tongue of the receiver (estimated to be equivalent to the surface covered by three swabs).

### Ethics approval

The research proposed in this study was evaluated on 25 June 2012 by the Central Committee on Research Involving Human Subjects (CCMO), The Hague, The Netherlands. According to the chair of the CCMO, the treatment or forms of behavior involved in this study were not intrusive or deviant from daily practices to the extent that they needed approval from the Medical Ethical Research Committee, according to the Medical Research Involving Subjects Act. All subjects provided written consent prior to the execution of the study.

## Availability of supporting data

The sequence data are available in the European Nucleotide Archive (ENA) under accession number PRJEB6781 (http://www.ebi.ac.uk/ena/data/view/PRJEB6781).

## Abbreviations

MH index: Morisita-Horn index; OTU: operational taxonomic unit in this study based on 97% percent similarity threshold for DNA sequence identity at the V5-V7 locus (16S rRNA).

## Competing interests

The authors declare that they have no competing interests.

## Authors’ contributions

MC analyzed the data, carried out the statistical analysis, and generated all additional files. WvE performed the DIC microscopy. GR analyzed the data and designed Figure 1. RK designed and coordinated the study, analyzed the data, and drafted the manuscript. All authors read and approved the final manuscript.

## Supplementary Material

Additional file 1 **Sequence data.** Data sheet containing OTU number, number of reads per OTU, taxonomic assignment down to the genus level, the V5-V7 amplicon DNA sequence, sample IDs, and metadata: number of reads for each of the 3000 OTU’s for each sample. Click here for file

Additional file 2 **Similarity indices.** Similarity of oral microbiota is indicated by the average index for similarity in community structure (Morisita-Horn index, Bray-Curtis index, StructPearson, the Yue and Clayton theta, Species Profile, and Spearman similarity coefficients) for the saliva and tongue samples from (i) the same individuals before and after kissing, (ii) the two different individuals within a couple before kissing, (iii) the two different individuals within a couple after kissing, and (iv) different, unrelated individuals before kissing. Click here for file

Additional file 3 **Reported kiss history analysis.** Correlations between self-reported intimate kiss frequency or time past since last kiss and microbiota similarity indices. Click here for file

Additional file 4 **Bacterial transfer calculation.** Estimation of bacterial transfer after an intimate kiss by tracking probiotic marker bacteria. Click here for file

Additional file 5 **DIC micrographs.** Differential interference contrast micrographs of tongue, saliva and yoghurt drink. Click here for file
