# Supplementary material for: Shaping the oral microbiota through intimate kissing
Source: Microbiome. 2014 Nov 17;2:41. doi: 10.1186/2049-2618-2-41 (PMC4233210; doi:10.1186/2049-2618-2-41)
Supplement: Additional file 5 — DIC micrographs. Differential interference contrast micrographs of tongue, saliva and yoghurt drink. [file 2049-2618-2-41-S5.pptx]

## Slide 1
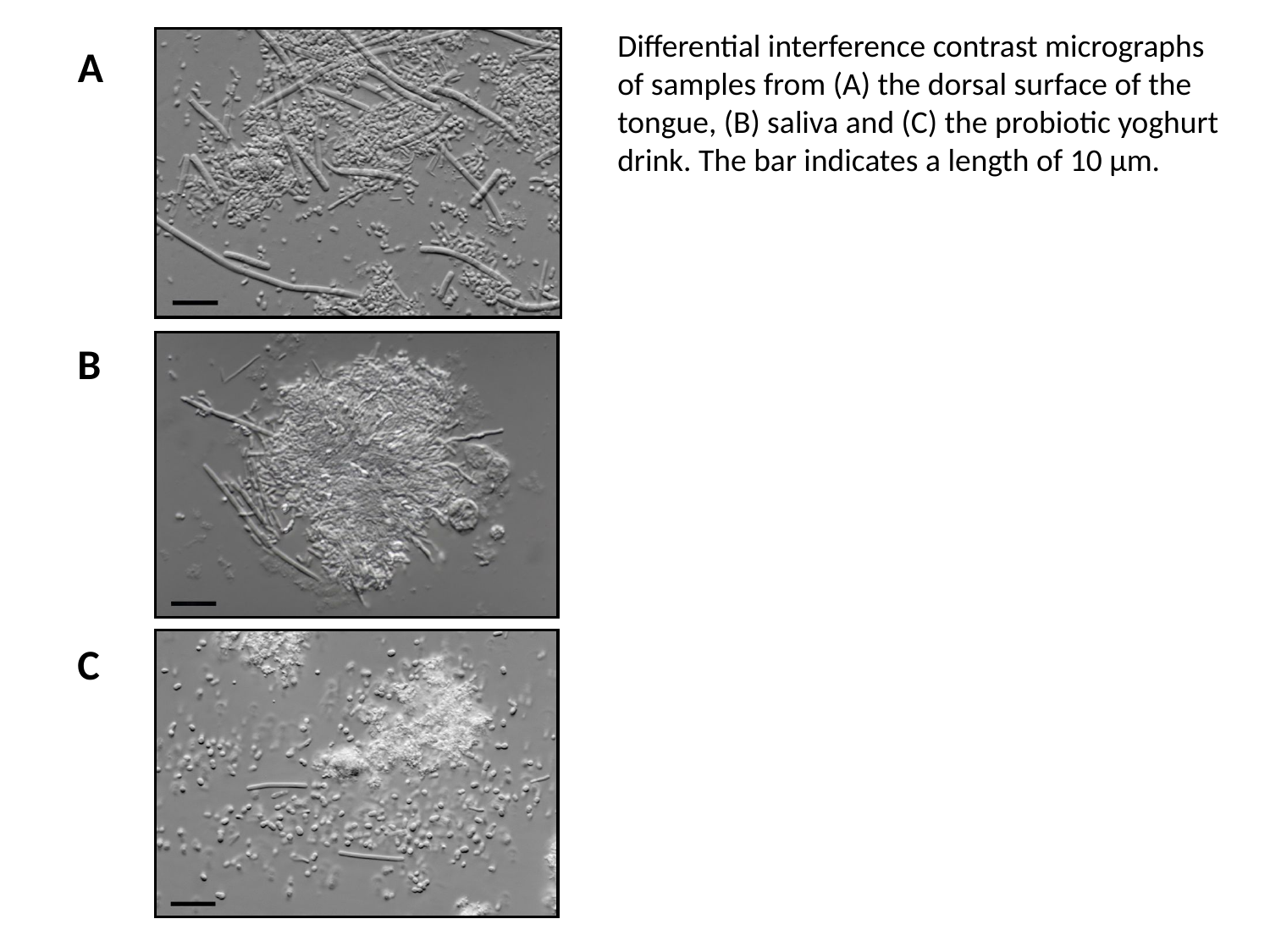

Differential interference contrast micrographs of samples from (A) the dorsal surface of the tongue, (B) saliva and (C) the probiotic yoghurt drink. The bar indicates a length of 10 µm.
A
B
C
